# Latent tuberculosis infection and kidney transplantation

**DOI:** 10.1590/2175-8239-JBN-2021-E008

**Published:** 2021-09-20

**Authors:** Felipe Francisco Tuon

**Affiliations:** 1 Pontifícia Universidade Católica do Paraná, Faculdade de Medicina, Laboratório de Doenças Infecciosas Emergentes, Curitiba, PR, Brasil.

The incidence rate of tuberculosis (TB) in kidney transplant (KT) recipients is higher than that in the normal population, and it is associated with unfavorable outcomes including graft loss and mortality^1^. Screening for latent TB (LTBI) in KT candidates can be accomplished by a thorough clinical history and physical examination, chest radiography, and specific tests, including tuberculin skin test (TSTs) and/or interferon gamma release assays (IGRAs). World Health Organization guidelines do not advocate ​​for one test over others, especially in immunocompromised patients^2^. IGRAs have a better predictive power than TSTs for LTBI, regardless of patient profile. However, the disagreement between TSTs and IGRAs can be high, and dual testing is recommended in certain populations, including patients with human immunodeficiency virus infection and those who have undergone solid organ transplant, as discussed below^3^.

The dormant bacilli associated with LTBI are commonly found in pulmonary granulomas, a risk factor for the development of TB after immunosuppressive therapy. However, there is evidence that patients harbor tuberculosis bacilli in extrapulmonary sites, including the kidneys, which poses a risk to KT recipients. This is important for the diagnosis of LTBI in potential living donors (LDs).

In a recent publication by Meinerzet al. (2021)^4^ in the *Brazilian Journal of Nephrology*, 116 KT recipients and 25 LDs were screened for LTBI by TSTs and IGRAs. IGRA results were positive in 30.2% of KT recipients, but only 18.1% had positive TST results. First, this positivity rate is significant and compatible with regional epidemiology, as shown in an older Brazilian study^5^. Second, the study shows that IGRA positivity is higher than TST positivity. Interestingly, several risk factors were associated with LTBI, including a past TB history, residual lesions on chest radiography, and preexisting diabetes. It is important to note that several studies have failed to identify risk factors that contribute to a positive test in solid organ transplant candidates, indicating that LTBI screening needs to be universal, regardless of the regional incidence of TB^6^. This is because there is no agreement on which test to use, as the sensitivity of TSTs and IGRAs can vary depending on the incidence of TB in the population.

In Japan, the incidence of TB is low, and the estimated prevalence of LTBI based on IGRAs is 3.7% in KT recipients. These results are consistent with the IGRA positivity rate in the general Japanese population, even under immunosuppressive therapy^7^. The prevalence in Brazil is approximately 10 times higher. Nevertheless, it is important to clarify that, even in regions with highly prevalent, as demonstrated by Meinerz et al. (2021)^4^, universal treatment of LTBI is not recommended.

All potential LDs and KT recipients must be screened for active TB prior to LTBI treatment following a positive IGRA or TST. LTBI treatment is given as monotherapy with isoniazid. Treating TB with only one drug can lead to resistance, delay TB diagnosis, and increase the risk of graft loss and mortality due to disease dissemination. In a study of 1150 KT recipients, 3.2% of the patients with positive IGRAs without isoniazid treatment developed TB, while none of the patients with positive IGRAs with isoniazid treatment developed TB^8^. This study is based on a larger patient sample but shows a similar trend to the study by Meinerz et al (2021)^4^.

The current publication is important because there are limited data on the sensitivity and specificity of IGRAs and TSTs for LTBI screening in kidney transplant recipients, especially in low-to-middle income countries. Data from studies of patients receiving hemodialysis are expected to yield more false-positive, false-negative, and indeterminate IGRA results^9^. However, IGRAs are superior to TSTs in hemodialysis patients and are found to be more sensitive than TSTs in diagnosing LTBI in patients requiring renal transplantation^10^.

The agreement between IGRAs and TSTs results is low, and this can render interpretation and management difficult for transplanters. An interesting advantage of IGRAs is its serial evaluation, as in the follow-up, both the values ​​and results may vary, and not treating LTBI when TST and IGRA results are discrepant is a conservative aproach^11^. This is because in several studies, including with pre-KT recipients, TSTs have shown a greater sensitivity than IGRAs^12^. Another important discussion is about LTBI prevalence in KT recipients being twice as high as in KT candidates, reinforcing the importance of continuous surveillance with semestral IGRAs for at least two years after KT^13^.

As the authors inform, unfortunately, the cost of IGRAs is considerably high for large-scale implementation, but are cost-effective in KT recipients and should be funded by the Public Health System, since rapid molecular tests are already funded^14^.

In summary, IGRA are superior to TSTs, but the disagreement between test results is high, and the false negative rate of IGRAs should not be overlooked. Therefore, double testing is an interesting strategy. However, in low-resource settings, TST is the only available test for LTBI diagnosis, but IGRAs should be chosen if available and if given the option of only one test.
